# Porcine Endogenous Retroviruses: Quantification of the Viral Copy Number for the Four Miniature Pig Breeds in China

**DOI:** 10.3389/fmicb.2022.840347

**Published:** 2022-03-15

**Authors:** Tao-feng Lu, Bo Sun, Tai-yong Yu, Yan-jun Wu, Jie Zhou, Shu-guang Wu

**Affiliations:** ^1^Institute for Laboratory Animal Research, Guizhou University of Traditional Chinese Medicine, Guiyang, China; ^2^The First Clinical Medical College, Jinan University, Guangzhou, China; ^3^College of Animal Science and Technology, Northwest A&F University, Yangling, China; ^4^Shanghai Laboratory Animal Research Center, Shanghai, China

**Keywords:** PERV, miniature pig model, ddPCR, copy number, quantification

## Abstract

Domestic pigs has served not only as one of the most important economy livestock but also as ideal organ-source animals owing to similarity in anatomy, physiology, and organ size to humans. Howerer, the barrier of the cross-species transmission risk of porcine endogenous retrovirus (PERVs) blocked the pig-to-human xenotransplantation. PERVs are integrated into pigs’ genomes and cannot be eliminated by designated or specified pathogen-free breeding. PERVs are an important biosafety issue in xenotransplantation because they can be released from normal pig cells and infect human cells *in vitro* under certain conditions. Screening and analyzing the presence of PERVs in pig genome will provide essential parameters for pig breed sources. In China, four miniature pig breeds, such as Guizhou miniature pig (GZ), Bama miniature pig (BM), Wuzhishan miniature pig (WZS), and Juema miniature pig (JM), were the main experimental miniature pig breeds, which were widely used. In this study, PCR was performed to amplify *env-A*, *env-B*, and *env-C* for all individuals from the four breeds. The results revealed that PERV *env-A* and *env-B* were detected in all individuals and the lowest ratios of PERV *env-C* was 17.6% (3/17) in the GZ breed. Then, PERV *pol* and *GAPDH* were detected using the droplet digital PCR (ddPCR) method. As the reference of *GAPDH* copy number, the copy numbers of PERVs were at the median of 12, 16, 14, and 16 in the four miniature pig breeds (GZ, BM, WZS, and JM), respectively. Furthermore, the copy number of the PERV *pol* gene in many organs from the GZ breed was analyzed using ddPCR. The copy numbers of PERV *pol* gene were at the median of 7 copies, 8 copies, 8 copies, 11 copies, 5 copies, 6 copies, and 7 copies in heart, liver, spleen, lung, kidney, muscle, and skin, and the maximum number was 11 copies in the lung. The minimum number was 5 copies in the kidney as the reference of *GAPDH*. These data suggest that GZ breed has the lower PERVs copy number in the genome, and may be an ideal organ-source miniature pig breed for the study of the pig-to-human xenotransplantation.

## Introduction

Transplantation is the most effective therapy for patients in treating organ failure. Nevertheless, the shortage of organs for transplantation is a major problem. Therefore, seeking animals as the organ source for human transplantation has become a new choice to solve this problem (Niu et al., 2021). Pig (*Sus scrofa*) has served not only as one of the most important economy livestock but also as an important model animal used in many areas of medical research. Although non-human primates have higher similarities in physiology, immune system, and anatomy to humans, pigs are now more favored due to their multiple advantages, such as cheaper, rapid population propagation, the significantly lower maintenance cost, and the adequate adult organ size (Lu et al., 2019; Zhang et al., 2019).

While porcine organs are considered promising, their use has been checked by concerns about the cross-species transmission of zoonotic pathogenic microorganisms. Porcine endogenous retroviruses (PERVs) cause severe diseases such as immune deficiencies and tumors in their hosts (Hartmann, 2012; Denner and Young, 2013). PERVs are integrated into pigs’ genomes, and they can be released from normal pig cells and can infect human cells *in vitro* under certain conditions (Guell, 2020; Denner, 2021), which is why PERVs are still considered a risk for xenotransplantation using pig cells, tissues, and organs (Kruger et al., 2020). Furthermore, unlike many other potentially zoonotic pig microorganisms, PERVs cannot be eliminated by designated or specified pathogen-free (DPF or SPF) breeding.

Porcine endogenous retroviruses are γ-retroviruses. Like other related γ-retroviruses, such as the feline leukemia virus (FeLV) (Denner and Young, 2013), koala retrovirus (KoRV) (Xu and Eiden, 2015), and the murine leukemia virus (MuLV) (McMichael et al., 2019), PERVs have two main mRNAs: a full-length mRNA encoding the core proteins (Gag, group-specific antigen) and the enzymes such as RT, RNase H and protease, and a spliced mRNA encoding the two envelopes (Env) proteins (Hirata et al., 2019). In pigs, three types of PERVs have been described: PERV-A and PERV-B, found in all pigs, and PERV-C, which are found in many, but not all, pigs (Denner and Tonjes, 2012; Denner, 2021). PERV-A and PERV-B can infect cells from humans and many other species, while PERV-C can only infect pig cells (Denner and Tonjes, 2012; Kruger et al., 2020). In addition, differences in PERV expression in different organs at the mRNA and the protein level have also been observed (Denner, 2016). Furthermore, recombinants between PERV-A and PERV-C (PERV-A/C) were found in living pigs that can also infect human cells (Kono et al., 2020; Kruger et al., 2021).

There are over 730 distinct pig breeds worldwide (Ai et al., 2015), different pig breeds have different copy numbers of PERVs, ranging from 1 to >100 (Denner, 2016, 2021). Therefore, estimating the exact copy number of PERVs is important for organ transplantation to eradicate the PERVs in the pig genome (Kimsa-Dudek et al., 2015). The quantification of PERV copy numbers is highly dependent on the technique used (Jung et al., 2010; Kaulitz et al., 2011; Fiebig et al., 2018). The most accurate method of detecting PERV copy numbers is digital droplet PCR (ddPCR). The method is a new technology utilizing a water–oil emulsion droplet system. Following ddPCR, each droplet is analyzed to determine the fraction of PCR-positive droplets in the sample of both PERV and the reference gene (Fiebig et al., 2018; Kruger et al., 2020). For the research of the genome-wide inactivation of PERV, this study aim to quantify the exact copy number of PERVs in China’s major miniature pig breeds using the ddPCR method, and then a better miniature pig breed will be confirmed for the study of PERV-free pigs. This study will screen out an ideal organ-source miniature pig breed for the pig-to-human xenotransplantation.

## Materials and Methods

### Animals

Four miniature pig breeds from China were used in this study. Healthy animals were selected for the study. Ear marginal tissue samples from 14 healthy Bama miniature pigs (BM) in closed colony were obtained from Guangxi University. Ear marginal tissue samples from 18 healthy Wuzhishan miniature pigs (WZS) in a closed colony were obtained from Guangdong Laboratory Animals Monitoring Institute. Ear marginal tissue samples from 15 Juema miniature pigs (JM) in a closed colony were obtained from Northwest Agriculture & Forestry University. Blood tissue samples from 17 Guizhou miniature pigs (GZ) in a closed colony were obtained from Guizhou University of Traditional Chinese Medicine. The heart, liver, spleen, lung, kidney, muscle, and skin tissue samples from three GZ pigs were obtained to analyze the PERV copy numbers in different organs. All experimental procedures used in this study were carried out following the Experimental Animal Management Regulations (amendment on 1 March 2017, China).

### Genomic DNA and Total RNA Isolation

DNA was extracted from the ear marginal and blood tissue samples using the phenol–chloroform extraction method. According to the manufacturer’s protocol, the extracted DNA was quantified using the NanoDrop LITE spectrophotometer (Thermo Fisher Scientific, United States).

For RNA isolation, the organ tissues (heart, liver, spleen, lung, kidney, muscle, and skin) were snap frozen immediately in liquid nitrogen after harvest and store at −80°C until extraction. According to the manufacturer’s instructions, total RNA was extracted from the tissues using TRIzol reagent (Invitrogen, Carlsbad, CA, United States). The extracted RNA samples were quantified using a NanoDrop LITE spectrophotometer (Thermo Fisher Scientific, United States).

### Amplification of PERV Proviral DNA by Classical PCR

The classical PCR method was employed to amplify the *env* region of PERV (*PERV env*), all PERV types (PERV-A, PERV-B, and PERV-C) were detected, and the sequences of primers are described in Table 1. The PCR reaction was performed using 2× EasyTaq PCR SuperMix (TransGen Biotech, Beijing, China). The PCR amplification mixture was initially incubated at 94°C for 5 min, and then 30 cycles of denaturation at 94°C for 30 s, corresponding annealing temperature (58-60°C) for 40 s, elongation at 72°C for 40 s and final elongation at 72°C for 7 min. The PCR product was visualized by 1.5% agarose gel electrophoresis and UV transillumination.

**TABLE 1:** Primers and probes for PERV detection.

Primer, probe	Sequence	Accession number	Position (nt–nt)	Reference
PERV-EnvA-F	5′-TGGAAAGATTGGCAACAGCG-3′	Y12238	742–761	Fiebig et al., 2018
PERV-EnvA-R	5′-AGTGATGTTAGGCTCAGTGG-3′		1,101–1,082	
PERV-EnvB-F	5′-TTCTCCTTTGTCAATTCCGG-3′	Y12239	1,376–1,395	Fiebig et al., 2018
PERV-EnvB-R	5′-TACTTTATCGGGTCCCACTG-3′		1,639–1,620	
PERV-EnvC-F	5′-CCCCAACCCAAGGACCAG-3′	AM229312	9,601–9,618	Kaulitz et al., 2013
PERV-EnvC-R	5′-AAGTTTTGCCCCCATTTTAGT-3′		9,692–9672	
PERV-pol-F	5′-CGACTGCCCCAAGGGTTCAA-3′	HM159246	3,568–3,587	Yang et al., 2015
PERV-pol-R	5′-TCTCTCCTGCAAATCTGGGCC-3′		3,803–3,783	
PERV-pol-probe	5′-[FAM]CACGTACTGGAGGAGGGTCACCTG[BHQ1]-3′		3,678–3,655	
Pig-GAPDH-F	5′-CCGCGATCTAATGTTCTCTTTC-3′	396823	3,951–3,970	Yang et al., 2015
Pig-GAPDH-R	5′-TTCACTCCGACCTTCACCAT-3′		4,022–4,001	
Pig-GAPDH-probe	5′-[HEX]CAGCCGCGTCCCTGAGACAC[BHQ1]-3′		3,991–3,972	

### Droplet Digital PCR

Droplet Digital PCR was performed according to the manufacturer’s instructions (Bio-Rad, Hercules, CA, United States) using a QX100 droplet generator and a QX100 droplet reader (Bio-Rad). Purified genomic DNA (100 ng genomic DNA) was digested with *Mse*I (New England Biolabs, United States) (20U) at 37°C for 1 h, and the restriction enzyme was heat-inactivated at 65°C for 20 min. The digested DNA samples were diluted into 5 ng/μL for the ddPCR reaction. The ddPCR mix consisted of 10 μL 2× ddPCR Master mix, 1.8 μL of each 10 μM target primers (Table 1), 0.5 μL of each 10 μM probes (FAM/HEX), 1.8 μL digested DNA (5 ng/μL) and water to a total volume of 20 μL. The following cycling conditions were used: 10 min initial enzyme activation at 95°C, 30 s denaturation at 94°C, 1 min annealing and extension at 59°C (45 cycles) and final 10 min enzyme deactivation at 98°C using a Master cycler ProS (Eppendorf, Hamburg, Germany). The temperature ramp rate was 2°C per second. After ddPCR amplification, the concentration (copies/μL) of the PERV *pol* gene and porcine *GAPDH* were obtained. The ratio of the PERV copy number was calculated using porcine *GAPDH* as the reference gene to detect the exact copy number of PERV.

For examining PERV *pol* gene expression in the seven organs (heart, liver, spleen, lung, kidney, muscle, and skin) from the GZ breed, the extracted RNA samples were reversed transcription into cDNA using a reverse transcription system (TransGene Biological Science & Technology Company, Beijing, China). The cDNA samples were diluted into 5 ng/μL for the ddPCR reaction as mentioned above. After ddPCR amplification, the concentration (copies/μL) of the PERV *pol* gene and porcine *GAPDH* were obtained. The ratio of the PERV copy number was calculated using porcine *GAPDH* as the reference gene to detect the exact copy number of PERV.

### Ethical Statement

Animal breeding and care and all experiments were performed following “Regulations for the administration of affairs concerning experimental animals of China (CNAS-CL60).” All experimental procedures used in this study were carried out following the Experimental Animal Management Regulations (amendment on 1 March 2017, China).

### Statistics

Statistical analysis was carried out using GraphPad Prism version 5.01 (GraphPad Software, Inc., San Diego, CA, United States),^1^ and conducted by one-way ANOVA with Tukey test for multiple comparisons. *P* values < 0.05 were considered to be statistically significant.

## Results

### Screening for PERV Proviral DNA in the Four Miniature Pig Breeds

**FIGURE 1:**
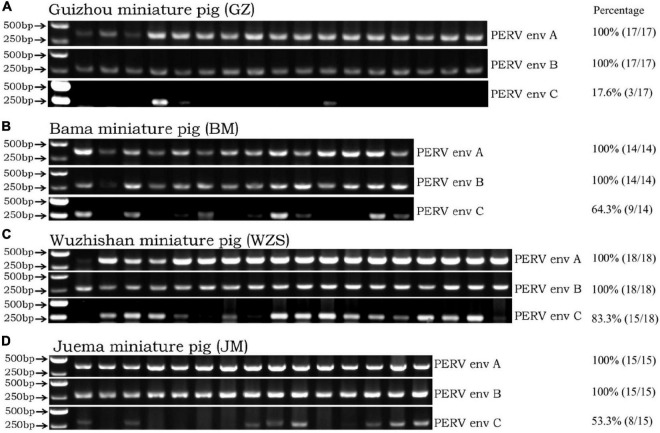
The classical PCR method detects env-A, env-B, and env-C genes in the four miniature pig breeds. Panel **(A)** was the detection result for the GZ breed. Panel **(B)** was the detection result for the BM breed. Panel **(C)** was the detection result for the WZS breed. Panel **(D)** was the detection result for the JM breed. The quantification data of the prevalence of PERV *env* A, B, C in each pig breeds was listed on the right.

PERV-A and PERV-B were found by PCR using specific primers for the PERV-*env A* and PERV-*env B* sequences in the four Chinese miniature pig breeds. All four Chinese miniature pig breeds were found positive for PERV-*env A* and PERV-*env B*. Therefore, their prevalence was 100% (Figure 1). When the four Chinese miniature pig breeds were screened for PERV-*env C*, the ratios of PERV-*env C* was 17.6% (3/17) in the GZ breed, the ratio was 64.3% (9/14) in the BM breed, the ratio was 83.3% (15/18) in the WZS breed, and the ratio was 53.3% (8/15) in the JM breed. These PCR results are shown in Figure 1.

### Determination of PERV Copy Numbers in Four Miniature Pig Breeds From Chinese

**FIGURE 2:**
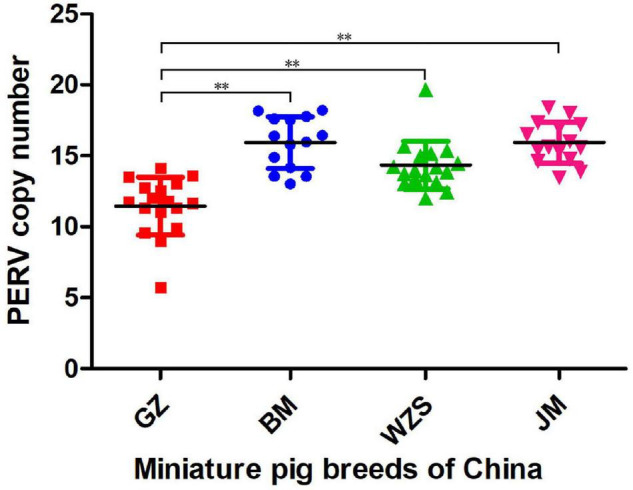
The copy number of PERV in the four different miniature pig breeds from China is based on the ddPCR method. Each point indicates one animal. The median and the standard deviations are shown. Guizhou miniature pig (GZ, red), Bama miniature pig (BM, blue), Wuzhishan miniature pig (WZS, green), and Juema miniature pig (JM, pink). The symbols of significant difference between comparisons of different pig breeds was marked (**P < 0.01).

Using a ddPCR with a probe corresponding to a *pol* sequence (Table 1) highly conserved among all PERVs and using the porcine *GAPDH* as a reference, the total copy number of PERV was measured. In the 17 GZ breed samples, PERV copy numbers between 6 and 14 were found with a median of 12 copies and a standard deviation of 2.0. In the 14 BM breed samples, PERV copy numbers between 13 and 18 were found with a median of 16 copies and a standard deviation of 1.8. In the 18 WZS breed samples, PERV copy numbers between 12 and 20 were found with a median of 14 copies and a standard deviation of 1.6. In the 15 JM breed samples, PERV copy numbers between 13 and 18 were found with a median of 16 copies and a standard deviation of 1.5. These results are shown in Figure 2. In summary, in the four Chinese miniature pig breeds, the PERV copy number varied between 6 and 20, significantly lower than the ratio of 46 in PK15 cells using *GAPDH* as a reference gene (Fiebig et al., 2018). Between the four Chinese miniature pig breeds, the differences between pigs in GZ and BM (*P* < 0.001), GZ and WZS (*P* < 0.001), and GZ and JM (*P* < 0.001) were significant (Figure 2).

### PERV Copy Numbers in Different Organs From the GZ Breed

**FIGURE 3:**
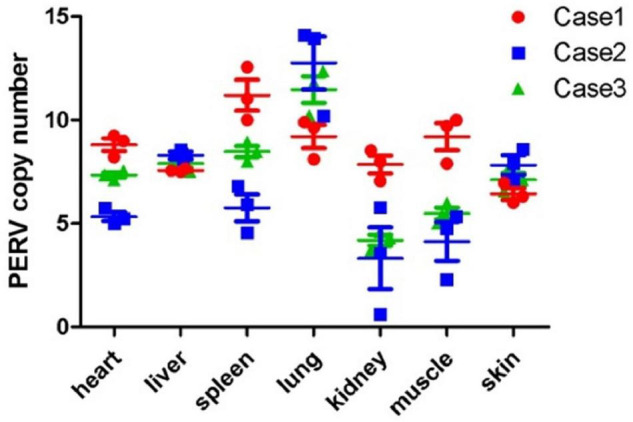
The copy number of PERV in the different organs from the GZ breed is based on the ddPCR method. Each point indicates one test. The median and the standard deviations are shown. Three GZ breed cases were tested.

The PERV copy number in domestic pigs is different in different organs of a single animal. Organs from the GZ breed were analyzed to analyze whether the same situation can be observed in Chinese miniature pig breeds. For testing the transcriptional activity of PERV, we tested the PERV *pol*-gene copy number in the seven organs (heart, liver, spleen, lung, kidney, muscle, and skin) from three pigs (case 1, case 2, and case 3) using ddPCR. The results are shown in Figure 3. The results revealed that the copy numbers of PERVs were at the median of 7, 8, 8, 11, 5, 6, and 7 in the heart, liver, spleen, lung, kidney, muscle, and skin. In case 1, the PERV copy number varied between 6 and 11 with a median of 9, and the maximum number was 11 copies in the spleen, and the minimum number was 6 copies in the skin. In case 2, the PERV copy number varied between 3 and 13 with a median of 7, and the maximum number was 13 copies in the lung, and the minimum number was 3 copies in the kidney. In case 3, the PERV copy number varied between 4 and 11 with a median of 7, and the maximum number was 11 copies in the lung, and the minimum number was 4 copies in the kidney. In the three pigs (case 1, case 2, and case 3), the median of PERV copy number for all organs was 8 copies, and the maximum number was 11 copies in the lung, and the minimum number was 5 copies in the kidney.

## Discussion

Since the 1980s, the Guizhou University of Traditional Chinese Medicine has cultivated a closed colony of an experimental miniature pig breed named Guizhou miniature pig (*Sus scrofa domestica var. mino Guizhounensis Yu*.) in Guiyang, China. After more than 30 years of breeding, the Guizhou miniature pig colony gradually became the experimental miniature pig population with stable genetic properties and well-controlled microbiological indicators. These pigs are small, gentle in disposition, have strong infertility, good adaptability, and are strongly resistant to many diseases. These pigs were also widely used in studying biomedical and traditional Chinese medicine. However, there is no study investigating PERV classification in Guizhou miniature pigs to determine whether the pig breed is a suitable donor source for xenotransplantation.

In China, the miniature pig breeds, such as Wuzhishan miniature pig inbred line from Hainan province, Bama miniature pig from Guangxi province, Guizhou miniature pig from Guizhou province, Juema miniature pig from Shaanxi province, and Banna miniature pig inbred line from Yunnan province, were the main experimental miniature pig breeds, which were widely used in the life science researches. However, the system research about the copy number of PERVs in the genome was also not mentioned in China’s major miniature pig breeds using ddPCR. For quantification study of the PERV copy number, only one study (Ma et al., 2010) revealed that the copy numbers of PERV from WZS genomic DNA ranged from 1.38 ± 0.33 to 14.23 ± 3.31, while the copy numbers of PERV from BM genomic DNA, 2.96 ± 0.76 to 58.46 ± 3.50 using real-time qPCR with SYBR Green I method.

Many researchers in other countries are also actively seeking donors for xenotransplantation. It has been reported that some Gottingen mini pigs contain PERV-A and PERV-B, and some contain PERV-C, but no PERV-A/C recombinant has been detected (Schuurman and Patience, 2013). A large white swine herd confirmed the ubiquity of PERV-A and PERV-B sequences, whereas PERV-C was absent in some individuals (Bosch et al., 2000). These studies revealed that the distribution of PERV was different in different pig breeds. This study selected four miniature pig breeds (BM, WZS, GZ, and JM) from china, and the prevalence and expression of PERVs were analyzed. All tested animals harbored PERV-A and PERV-B in their genomes, and the GZ breed has the lowest ratios of PERV-C (17.6%) in the four miniature pig breeds. The prevalence of PERV-C was different in the four miniature pig breeds and the other Chinese pig breeds, such as Diannan small-eared pigs (Zhang et al., 2020), Daweizi pigs (Xing et al., 2006), and Ningxiang pigs (Xing et al., 2009). For the PERV copy number of the four miniature pig breeds, the GZ breed also has the lowest copy number (median as 6), and the differences were significant between the other breeds and the PK15 cell line (Fiebig et al., 2018).

The copy number of PERV cannot be determined correctly, not knowing the copy number of the reference gene, and the copy number of reference genes such as *GAPDH* and *ACTB* seems to vary in the same sample. In one study, the researcher compared the apparent copy number of PERVs in PK15 cells using *GAPDH* or *ACTB* as reference genes. The PERV copy number was 46 in PK15 cells using *GAPDH* as a reference gene and was 30 determined using *ACTB*. The result suggested that two *GAPDH* and three *ACTB* genes are present in the pig genome. This study measured the concentration (copies/μL) of the PERV *pol* gene and porcine *GAPDH* using the ddPCR method. The ratio of the PERV copy number was calculated using porcine *GAPDH* as the reference gene. So, the exact copy number of PERV can be calculated according to the *GAPDH* genes numbers in the pig genome. Concretely speaking, the exact copy number of PERV in pig genome is twice the radio.

In summary, we have quantified the exact copy number of PERVs in China’s major miniature pig breeds using the ddPCR method, and measured the PERV copy numbers in different organs from the GZ breed. These data suggest that GZ breed has the lower PERVs copy number in the genome, and may be an ideal organ-source miniature pig breed for the study of the pig-to-human xenotransplantation.

## Data Availability Statement

The original contributions presented in the study are included in the article, further inquiries can be directed to the corresponding authors.

## Ethics Statement

The animal study was reviewed and approved by the IACUC of Guizhou University of Traditional Chinese Medicine. The animal ethics approval number is Guizhou SYXK-2021-02. Written informed consent was obtained from the owners for the participation of their animals in this study.

## Author Contributions

T-FL and BS wrote the manuscript. T-FL and JZ prepared the graphs and table. T-FL and S-GW reviewed and edited the manuscript. T-YY and Y-JW collected the samples. All authors read and approved the final manuscript.

## Conflict of Interest

The authors declare that the research was conducted in the absence of any commercial or financial relationships that could be construed as a potential conflict of interest.

## Publisher’s Note

All claims expressed in this article are solely those of the authors and do not necessarily represent those of their affiliated organizations, or those of the publisher, the editors and the reviewers. Any product that may be evaluated in this article, or claim that may be made by its manufacturer, is not guaranteed or endorsed by the publisher.

## Footnotes

1
www.graphpad.com
